# Evaluating Post-Transplant Outcomes in Elderly Liver Recipients Over 70: A Propensity-Score Matching Analysis

**DOI:** 10.3389/ti.2025.15429

**Published:** 2025-11-24

**Authors:** Edoardo Prosperi, Enrico Prosperi, Matteo Serenari, Chiara Bonatti, Guido Fallani, Alberto Stocco, Giorgia Radi, Federica Mirici Cappa, Cristiana Laici, Antonio Siniscalchi, Maria Cristina Morelli, Matteo Ravaioli, Matteo Cescon

**Affiliations:** 1 Department of Medical and Surgical Sciences, Alma Mater Studiorum, University of Bologna, Bologna, Italy; 2 Hepatobiliary and Transplant Unit, IRCSS, Azienda Ospedaliero-Universitaria di Bologna, Bologna, Italy; 3 Internal Medicine Unit for the Treatment of Severe Organ Failure, IRCCS Azienda Ospedaliero-Universitaria di Bologna, Bologna, Italy; 4 Post-Surgical and Transplant Intensive Care Unit, Department of Digestive, Hepatic and Endocrine-Metabolic Diseases, IRCCS Azienda Ospedaliero-Universitaria di Bologna, Bologna, Italy

**Keywords:** MASLD, propensity score matching, liver transplant recipients, elderly recipients, MELD score

## Abstract

The rising prevalence of Metabolic Dysfunction-Associated Steatotic Liver Disease (MASLD) and Hepatocellular Carcinoma in the elderly population has increased the demand for liver transplantation (LT) in patients over 70 years. Advanced age, however, is still considered an independent risk factor. This study aims to evaluate post-transplant oucomes in patients aged over 70 years, traditionally viewed as an age limit for transplant. We retrospectively analyzed 584 LT recipients (36 aged ≥70, 548 aged <70). Viral cirrhosis was more frequent in the younger group (36.1% vs. 13.1%), while MASLD was more common in those over 70 (25% vs. 13.1%) (p = 0.013). Model for End-Stage Liver Disease (MELD) scores were lower in patients over 70 (13, IQR 9–17) compared to the younger group (15, IQR 10–23) (p = 0.032). Propensity score matching (3:1 ratio, without replacement) was performed based on MELD and cirrhosis etiology. After matching, no significant differences were found in postoperative outcomes, overall survival, or graft survival. Our findings demonstrate that carefully selected patients over 70 can achieve post-transplant outcomes comparable to younger patients. Advanced age alone should not be considered an absolute contraindication; instead, a comprehensive, multidimensional assessment is essential to identify suitable candidates.

## Graphical Abstract

**Figure:**
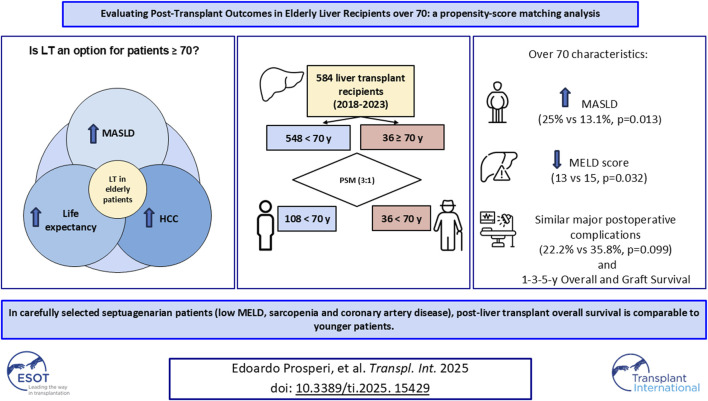

## Introduction

Liver transplant (LT) is the only curative treatment for end-stage liver disease (ESLD). Globally, along with a longer life expectancy, the prevalence of Metabolic Dysfunction-Associated Steatotic Liver Disease (MASLD) and Hepatocellular Carcinoma (HCC) is rising in patients older than 65 years old [1–3], resulting in an increased number of liver transplants performed in this group. This trend is observed both in the United States and in Europe [4–7]. Notably, older LT candidates often show multiple comorbidities, independently from liver disease, that negatively influence post-transplant outcomes [8, 9]. Despite the increasing amount of literature addressing this topic, findings remain inconclusive. Some studies have reported poorer outcomes associated with older age, including higher graft loss and reduced survival, in particular in patients with high Model For End-Stage Stage Liver Disease (MELD) scores [10, 11]. Conversely, other works have proved that liver transplantation in selected patients aged 70 and older is safe and feasible, showing graft survival and overall survival rates similar to younger cohorts [12–14] [15]. Importantly, the guidelines from American Association for the Study of Liver Diseases and the American Society of Transplantation do not consider age over 70 years old a contraindication to LT, in the absence of significant comorbidities [16].

This heterogeneity in published results probably reflects differences found in previous analyses in study design, patient selection and institutional practices.

Emerging evidence suggests that, rather than chronological age alone, the severity of comorbidities and liver disease and the general functional status of the patients represent more reliable prognostic indicators of post-transplant outcomes, underscoring the importance of careful selection in achieving favorable results.

We present our experience with LT recipients aged ≥70 years. This study aims to describe the characteristics of our cohort of recipients aged over 70 and evaluate their post-transplant outcomes in comparison to younger recipients.

## Materials and Methods

### Study Design and Setting

**FIGURE 1:**
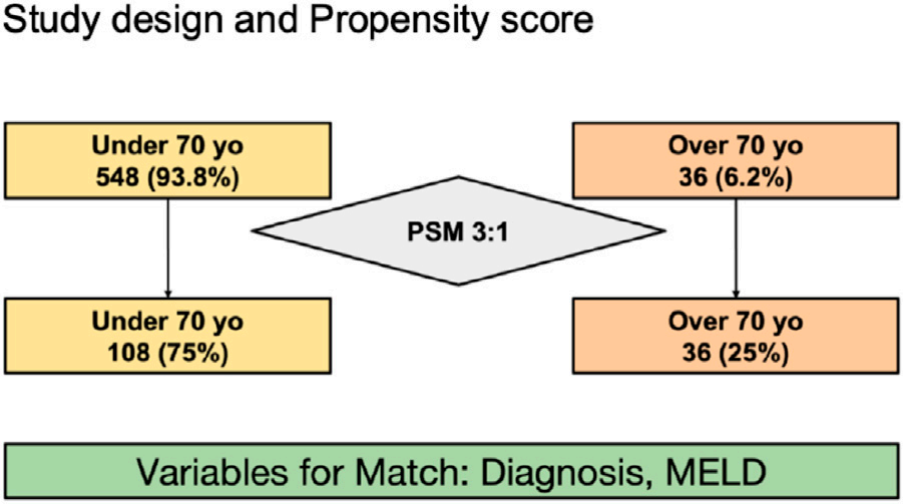
Study design and Propensity score.

Between January 2018 and November 2023, all consecutive adult recipients who underwent cadaver-donor LT in our center were included. We divided the entire cohort in two groups based on age at LT: younger than 70 years old and aged 70 years old or older (Figure 1).

This study was approved by the Institutional Review Board of our hospital.

### Data Collection and Clinical Variables

Data were collected prospectively and retrospectively analyzed. Recipient variables included demographics, MELD score at LT and urgent LT (defined, in our national practice, as MELD ≥28), indication for LT, comorbidities, and modified Charlson Comorbidity Index (high modified Charlson Comorbidity Index was defined as mCCI >1) [17].

Donor variables included demographics, donation type, whole or split liver graft, and cold ischemia time (CIT). Postoperative outcomes included complications (defined as major if Clavien-Dindo grade ≥2 [18]), Early Allograft Dysfunction (EAD) [19], Comprehensive Complication Index (CCI), 90-day mortality, 90-day readmission, patient survival and graft survival. During the study period, orthotopic liver transplantation was preferably performed with preservation of the inferior vena cava, with few cases performed with vena cava replacement. Over the years, an increasing number of Extended Criteria Donor (ECD) grafts were treated with Hypothermic Oxygenated perfusion (HOPE), which is at present our standard preservation technique with ECDs [20]. Regarding the cardiological assessment, all LT candidates underwent a standardized preoperative cardiological evaluation, including transthoracic echocardiography and Doppler ultrasound of the supra-aortic vessels. Coronary computed tomography angiography (CCTA) was indicated in patients presenting with one major or two minor cardiovascular risk factors.

Major risk factors included: age >50 years, diabetes mellitus, history of angina, myocardial infarction, stroke, transient ischemic attack, heart failure, or chronic kidney disease.

Minor risk factors included: smoking, hypercholesterolemia, hypertension, chronic alcohol consumption, and male sex >40 years or female sex >45 years.

When CCTA findings were inconclusive or suspicious for significant coronary artery disease, invasive coronary angiography was performed. Coronary stent placement was undertaken in the presence of significant stenosis, provided that the patient could complete at least 6 months of dual antiplatelet therapy before transplantation.

Stress echocardiography and/or myocardial scintigraphy were performed in selected cases, depending on patient suitability for the test and the availability of nuclear medicine facilities.

CT scans at the time of LT in the group over 70 years old were evaluated for assessing sarcopenia. Psoas Muscle Area (PMA) (mm^2^) is defined as the cross-sectional area of the left and right psoas muscles at the level of the third lumbar vertebra (L3). We employed as PMA cut-offs to define sarcopenia values < 1,561 mm^2^ in men and <1464 mm^2^ in women [21] The Psoas Muscle Index (PMI) was obtained as PMA (cm2) divided by square height (m^2^). We employed as PMI cut-offs to define sarcopenia values <340 mm^2^/m^2^ in men and <264 mm^2^/m^2^ in women [22].

### Multidisciplinary Board and Selection Process

Each candidate undergoes a final evaluation by a multidisciplinary board. Candidate assessment involves transplant surgeons, hepatologists, transplant anesthesiologists, radiologists, and psychologists. Additional evaluation by other specialists are requested in case of complex cases. Final eligibility is based on a comprehensive evaluation of the comorbidities, functional status, cognitive integrity, oncologic considerations (if applicable), and expected post-transplant benefit.

### Objectives

The primary endpoint of the study was to evaluate post-transplant mortality, overall patient survival and graft survival comparing recipients aged 70 or older with those younger than 70.

The secondary endpoint was to evaluate postoperative complications.

### Statistical Analysis

Categorical variables were reported as absolute numbers and percentages and compared using the Chi-square test or Fisher’s exact test, as appropriate. Continuous variables were expressed as medians with interquartile ranges (IQR) and compared using the Mann–Whitney U test. A two-tailed p-value <0.05 was considered statistically significant. Statistical analyses were performed using STATA 18.

Patients of both groups were matched using the propensity score method (PSM) as described by Rubin and Rosenbaum [23]. The propensity score for an individual was calculated given the covariates of LT indication and MELD score, the two variables that differed significantly between the two groups. A 3:1 matching was performed without replacement to minimize conditional bias (Figure 1). For each recipient in the younger group, an elderly recipient with a minimum difference on the propensity score was matched.

## Results

### Entire Cohort Characteristics

Within the cohort of 584 patients, the elderly group (n = 36, 6.2%) showed a median age of 71 (70–72 IQR), whereas the younger than 70 years group (n = 548, 93.8%) showed a median age of 58 (52–63 IQR) (Table 1).

**TABLE 1:** Preoperative and donor Parameters before and after PSM.

Preoperative parameters	Before PSM				After PSM			
	Under 70 Yo	Over 70 Yo	Total	p-value	Under 70 Yo	Over 70 Yo	Total	p-value
	(N = 548)	(N = 36)	(N = 584)		(N = 108)	(N = 36)	(N = 144)	
Recipient age, years	58 (52–63)	71 (70–72)	58 (53–64)	<0.001	56 (48–63)	71 (70–72)	61 (52–70)	<0.001
Recipient gender male	364 (66.4%)	20 (55.6%)	384 (65.8%)	0.183	67 (62.0%)	20 (55.6%)	87 (60.4%)	0.491
BMI	25 (23–28)	25 (22–28)	25 (23–28)	0.363	26 22.29	25 (23–29)	26 (22–29)	0.458
mCCI >1	67 (12.2%)	7 (19%)	113 (26%)	0.207	5 (6.8%)	7 (19.4%)	12 (10.9%)	0.045
Liver transplant indication								
Viral	198 (36.1%)	5 (13.9%)	203 (34.8%)	0.013	22 (20.4%)	5 (13.9%)	27 (18.8%)	0.633
Alcohol-related liver disease	126 (23.0%)	7 (19.4%)	133 (22.8%)		17 (15.7%)	7 (19.4%)	24 (16.7%)	
MASLD	72 (13.1%)	9 (25.0%)	81 (13.9%)		19 (17.6%)	9 (25.0%)	28 (19.4%)	
Other	152 (27.7%)	15 (41.7%)	167 (28.6%)		50 (46.3%)	15 (41.7%)	65 (45.1%)	
Hepatocellular carcinoma	224 (40.9%)	13 (36.1%)	237 (40.6%)	0.573	44 (40.7%)	13 (36.1%)	57 (39.6%)	0.623
TIPS	33 (6.0%)	1 (2.8%)	34 (5.8%)	0.418	4 (3.7%)	1 (2.8%)	5 (3.5%)	0.623
Portal hypertension	354 (83.9%)	18 (81.8%)	372 (83.8%)	0.798	56 (71.8%)	18 (81.8%)	74 (74.0%)	0.344
Cirrhosis related complications								
Ascites	255 (57.2%)	14 (63.6%)	269 (57.5%)	0.550	52 (60.5%)	14 (63.6%)	66 (61.1%)	0.785
Hepatic encephalopathy	172 (49.6%)	7 (38.9%)	179 (49.0%)	0.377	33 (47.8%)	7 (38.9%)	40 (46.0%)	0.498
Jaundice	210 (49.8%)	9 (40.9%)	219 (49.3%)	0.418	27 (34.6%)	9 (40.9%)	36 (36.0%)	0.587
Coagulopathy	237 (56.8%)	13 (59.1%)	250 (56.9%)	0.835	38 (48.7%)	13 (59.1%)	51 (51.0%)	0.39
Esophageal varices	109 (26.3%)	4 (18.2%)	113 (25.9%)	0.395	18 (23.4%)	4 (18.2%)	22 (22.2%)	0.605
Comorbidities								
Respiratory	99 (23.5%)	8 (32.0%)	107 (24.0%)	0.334	20 (23.8%)	8 (32.0%)	28 (25.7%)	0.411
Renal	79 (18.5%)	4 (15.4%)	83 (18.3%)	0.694	7 (8.2%)	4 (15.4%)	11 (9.9%)	0.286
Cardiological	213 (47.8%)	12 (54.5%)	225 (48.1%)	0.534	41 (47.7%)	12 (54.5%)	53 (49.1%)	0.565
Coronarography	17 (3.1%)	2 (5.5%)	19 (3.2%)	0.422	4 (3.7%)	2 (5.5%)	6 (4.1%)	0.63
Diabetes	143 (31.2%)	13 (46.4%)	156 (32.0%)	0.093	29 (31.9%)	13 (46.4%)	42 (35.3%)	0.159
MELD at LT	15 (10–23)	13 (9–17)	15 (10–23)	0.032	12 (10–15)	13 (9–17)	12 (9–16)	0.149
Urgent LT	90 (16.4%)	4 (11.1%)	94 (16.1%)	0.401	5 (4.6%)	4 (11.1%)	9 (6.25%)	0.164
Platelets (10^3^/mmc)	82.5 (49–138.5)	113 (65–149.5)	84 (50–139)	0.078	82.5 (49–138.5)	113 (65–149.5)	84 (50–139)	0.814
Sodium (mmol/L)	137 (134–139)	137.5 (134.5–139.5)	137 (134–139)	0.541	137 (134–139)	137.5 (134.5–139.5)	137 (134–139)	0.752
Albumin (g/dL)	3.5 (3–4)	3.7 (3.3–3.9)	3.5 (3–4)	0.2068	3.5 (3.1–4)	3.7 (3.3–3.9)	3.6 (3.2–4)	0.558
Previous abdominal surgery	334 (61.1%)	22 (61.1%)	356 (61.1%)	0.995	77 (72.0%)	22 (61.1%)	99 (69.2%)	0.222
Donor parameters								
Donor age, years	64 (49–76)	64.5 (56.5–75)	64 (50–76)	0.420	67 (48.5–78)	64.5 (56.5–75)	67 (52–78)	0.358
Donor gender male	307 (56.0%)	17 (47.2%)	324 (55.5%)	0.303	62 (57.4%)	17 (47.2%)	79 (54.9%)	0.288
Donor BMI	25 (23–28)	25 (23–28)	25 (23–28)	0.2	26 (23–29)	25 (23–29)	25 (23–29)	0.456
DCD	60 (10.9%)	5 (13.9%)	65 (11.1%)	0.587	22 (20.4%)	5 (13.9%)	27 (18.8%)	0.388
Extended criteria donor	465 (84.9%)	29 (80.6%)	494 (84.6%)	0.34	93 (86.1%)	29 (80.6%)	122 (84.7%)	0.422
Split donor graft	20 (3.65%)	0	20 (3.42%)	0.243	5 (4.6%)	0	5 (4.5%)	0.189
Donor bilirubim	0.78 (0.49–1.1)	0.49 (0.4–0.87)	0.75 (0.48–1.1)	0.242	0.7 (0.5–1.1)	0.5 (0.4–0.9)	0.7 (0.5–1.1)	0.22
Donor GOT (U/L)	31 (20–61)	28 (20–51)	31 (20–61)	0.693	37 (23–70)	28 (19–50)	34 (22–68)	0.436
Donor GPT (U/L)	25 (15–53)	22 (14–39)	24 (14–52)	0.699	34 (15–63)	21 (13–39)	30 (15–61)	0.401
Donor Sodium (mmol/L)	149 (144–156)	149 (144–161)	149 (144–156)	0.372	147 (142–155)	149 (144–161)	147 (142–156)	0.146
Cold ischemia time, minutes	390 (350–450)	372 (340–445)	390 (350–450)	0.203	380 (335–422)	372 (340–445)	375 (337–430)	0.818
HOPE	225 (41%)	18 (50%)	243 (41%)	0.292	38 (35.2%)	18 (50%)	56 (38.89%)	0.864

Continuous Values Expressed as Median (IQR), categorical values expressed as number (%). Abbreviations: mCCI, Modified Charlson Comorbidity Index; DCD, Donor after Cardiac Death; HOPE, Hypothermic Oxygenated Perfusion; GOT, Glutamic Oxaloacetic Transaminase; GPT, Glutamate Pyruvate Alanine Aminotransferase.

### Recipients’ Characteristics

The majority of patients were male, with a higher percentage in the younger group (66.4% vs. 55.6%, p = 0.183) without a statistically significant difference. Median BMI was 25 in both groups (p = 0.363) (Table 1). The two groups differed significantly in cirrhosis etiology and median MELD score. Viral cirrhosis was the most frequent indication in the younger group (n = 198, 36.1% vs. n = 5, 13.9%, p = 0.013). MASLD was more frequent in the elderly group (n = 72, 13.1% vs. n = 9, 25%, p = 0.013). The elderly group had a significantly lower median MELD score (15 vs. 13, p = 0.017).

Clinically significant portal hypertension was present in 81.8% of the elderly group and in 83.9% of the younger group (p = 0.798). Diagnosis of Hepatocellular Carcinoma (HCC) did not differ between elderly and younger groups (36.1% vs. 40.9%, p = 0.573).

#### Complications of Cirrhosis and Laboratory Values

There were no statistically significant differences in terms of cirrhosis-related conditions between the two populations (Table 1). Ascites was the most frequent complication of cirrhosis in both groups (63.9% vs. 57.2%, p = 0.550). Encephalopathy, jaundice, and esophageal varices were slightly less frequent in the elderly population, but without reaching statistical significance (38.9% vs. 49.6%, p = 0.377; 40.9% vs. 49.8%, p = 0.418; 18.2% vs. 26.3%, p = 0.95, respectively).

Presence of coagulopathy was similar between the two groups (59.1% vs. 56.8%, p = 0.835).

Sodium and albumin levels were comparable between the elderly and the younger groups [137.5 mmol/L IQR (134.5–139.5) vs. 137 mmol/L IQR (134–139), p = 0.54; 3.7 g/dL IQR (3.3–3.9) vs. 3.5 IQR (3–4), p = 0.206].

#### Comorbidities

Respiratory, renal and cardiological comorbidities, and diagnosis of diabetes were similar between the two groups (Table 1). Cardiological comorbidities were the most common in both groups and, although they did not show statistically significant difference, were slightly higher in the elderly group, as well as diabetes and respiratory comorbidities (54.5% vs. 47.8%, p = 0.534; 46.4% vs. 31.2%, p = 0.093; 32% vs. 23.5%, p = 0.334, respectively). Pre-transplant coronarography rate was comparable between groups (5.5% vs. 3.1%, p = 0.422). The rate of previous abdominal surgery was equal (61.1% vs. 61.1%, p = 0.995). Before PSM analysis, mCCI >1 was slightly higher in the elderly group compared to the younger group, but without reaching statistical significance (19% vs. 12.2%, p = 0.207).

#### Specific Cardiological Comorbidities and Sarcopenia of the Elderly Group

Regarding specific history of cardiological comorbidities, 2 patients had history of myocardial infarction treated with PCI (n = 2, 5.6%) and 2 patients had history of atrial fibrillation. Coronary computed tomography angiography (CCTA) was performed in 21 patients, accounting for 58.3% of the group. Median Coronary Calcium Score (Agatston score) was 56 (IQR 0–989) (Table 2).

**TABLE 2:** Comorbidities and Sarcopenia scores of Elderly recipients.

Comorbidity	Frequency (%) or median
Hypertension	17 (47.2%)
Dyslipidemia	14 (40.0%)
Smoking	20 (55.5%)
Non-hepatic malignancy	2 (5.6%)
Chronic obstructive pulmonary disease (COPD)	9 (25.0%)
History of myocardial infarction (MI)	2 (5.6%)
History of percutaneous coronary intervention (PCI)	2 (5.6%)
Atrial fibrillation (AF)	2 (5.6%)
Coronary computed tomography angiography (CCTA)	21 (58.3%)
Coronary calcium score (agatston)	56 (0–989)
Psoas muscle area, (mm^2^)	1,700 (1,300–2,390)
Male, (mm^2^)	2,130 (1,600–2,500)
Female, (mm^2^)	1,300 (1,000–1,600)
Psoas muscle index, mm^2^/m^2^	625 (445–801)
Male, mm^2^/m^2^	763 mm^2^/m^2^ (555–849)
Female, mm^2^/m^2^	470 mm^2^/m^2^ (410–625)
Sarcopenia (defined by PMA) (n,%)	13 (36.1%)
Male (n, %)	5 (22.73%)
Female (n, %)	8 (57.14%)
Sarcopenia (defined by PMI) (n,%)	0%

Continuous Values Expressed as Median (IQR), categorical values expressed as number (%).

In the elderly cohort, median PMA was 1,710 mm^2^ (IQR 1,300–2,390), lower in females than males (1,300 vs. 2,130 mm^2^). Median PMI was 6.25 mm^2^/m^2^ (IQR 4.45–8.01), also lower in females than males (4.70 vs. 7.63 mm^2^/m^2^). Using PMA cut-offs, 13 patients (36.1%) were classified as sarcopenic (8 females, 5 males), whereas no patients met sarcopenia criteria using PMI cut-offs.

### Donor Characteristics

There were no statistically significant differences between the elderly and the younger group regarding the donor’s characteristics (Table 1). Median donor age was 64.5 years (IQR 56.5–75) in the elderly group vs. 64 years in the younger group (IQR 49–76) (p = 0.420), median donor BMI was 25 in both groups (IQR 23–28) (p = 0.2). Most of the grafts were classified as Extended criteria donors in both groups (n = 29, 80.6% vs. n = 465.84.9%, p = 0.489). Rate of DCD donors were comparable between the 2 groups (13.9% vs. 10.9%, p = 0.587). Median cold ischemia time was slightly lower in the elderly group, but without reaching statistical significance (372 min vs. 390 min, p = 0.203).

The use of Hypothermic oxygenated perfusion (HOPE) was comparable between the elderly and the younger group (50% vs. 41%, p = 0.292). The number of LT with split graft was 20 (3.65%) in the younger group, compared to 0 (0%) in the elderly group, without reaching statistical significance (p = 0.243).

### Postoperative Outcomes, Graft and Overall Survival

There were no significant statistical differences between the elderly group and the younger group regarding postoperative parameters (Table 3). The rate of major complications was similar between the two groups (22.2% vs. 35.8%, respectively, p = 0.099), as was CCI (29.6, vs. 30.2, p = 0.526).

**TABLE 3:** Postoperative parameters before and after PSM.

Post-operative parameters	Before PSM				After PSM			
	Under 70 Yo	Over 70 Yo	Total	p-value	Under 70 Yo	Over 70 Yo	Total	p-value
	(N = 548)	(N = 36)	(N = 584)		(N = 108)	(N = 36)	(N = 144)	
Early allograft disfunction	154 (28.1%)	7 (19.4%)	161 (27.6%)	0.26	33 (30.6%)	7 (19.4%)	40 (27.8%)	0.197
Hepatic artery thrombosis	6 (1.1%)	0 (0.0%)	6 (1.1%)	0.528	1 (0.9%)	0 (0.0%)	1 (0.7%)	0.566
Re-transplantation^a^	21 (3.8%)	1 (2.8%)	22 (3.8%)	0.748	7 (6.5%)	1 (2.8%)	8 (5.6%)	0.401
Re-laparotomy	78 (14.7%)	4 (11.4%)	82 (14.5%)	0.84	11 (10.3%)	4 (11.4%)	15 (10.6%)	0.848
Biliary complication	55 (10.3%)	1 (2.9%)	56 (9.8%)	0.152	10 (9.3%)	1 (2.9%)	11 (7.7%)	0.213
Cardiac complication	50 (11.4%)	1 (4.5%)	51 (11.0%)	0.319	8 (9.4%)	1 (4.5%)	9 (8.4%)	0.464
Postoperative infection	225 (41.9%)	14 (40.0%)	239 (41.8%)	0.825	40 (38.1%)	14 (40.0%)	54 (38.6%)	0.841
Pneumonia	47 (10.5%)	4 (18.2%)	51 (10.9%)	0.261	7 (8.1%)	4 (18.2%)	11 (10.2%)	0.165
Acute cellular rejection	29 (5.4%)	0 (0.0%)	29 (5.1%)	0.158	7 (6.6%)	0 (0.0%)	7 (5.0%)	0.119
Readmission by 30 post-operative day	113 (26.0%)	7 (31.8%)	120 (26.3%)	0.548	23 (27.7%)	7 (31.8%)	30 (28.6%)	0.705
Clavien dindo >2	196 (35.8%)	8 (22.2%)	204 (34.9%)	0.099	35 (32.4%)	8 (22.2%)	43 (29.9%)	0.248
CCI	30.2 (20.9–48.3)	29.6 (20.9–38.35)	29.6 (20.9–47.6)	0.516	29.6 (20.9–39.6)	29.6 (20.9–38.4)	29.6 (20.9–39.5)	0.976
Failure to rescue	25 (4.5%)	1 (2.8%)	26 (4.5%)	0.615	5 (4.6%)	1 (2.8%)	6 (4.2%)	0.63
LOS (days)	16 (11–29)	14.5 (12–17)	16 (11–28)	0.289	15 (12–17)	14.5 (12–17)	15 (12–17)	0.738
Mortality^b^	28 (5.11%)	2 (5.6%)	30 (5.1%)	0.907	7 (6.5%)	2 (5.6%)	9 (6.2%)	0.842

Continuous Values Expressed as Median (IQR), categorical values expressed as number (%). Abbreviations: CCI, Comprehensive Complication Index; LOS, Length of Hospital Stay.

^a^ Re-Transplantation within 30 days post Liver Transplantation.

^b^ Mortality Within 90 Days post Liver Transplantation.

The rates of EAD, hepatic artery thrombosis, postoperative infection, biliary and cardiac complications, pneumonia and acute rejection were comparable between the two populations. The 90-day mortality rates were comparable between the two groups (5.6% vs. 5.11%, p = 0.907). Rates of re-transplantation within 90 days were similar across the elderly and the younger group (2.8% vs. 3.8%, p = 0.748).

The two patients who died within the first 90 postoperative days were female. Both had an mCCI >1, and neither had a history of coronary artery disease. However, they belonged to the lowest quartile for sarcopenia measurements, based on both PMA and PMI scores.

**FIGURE 2:**
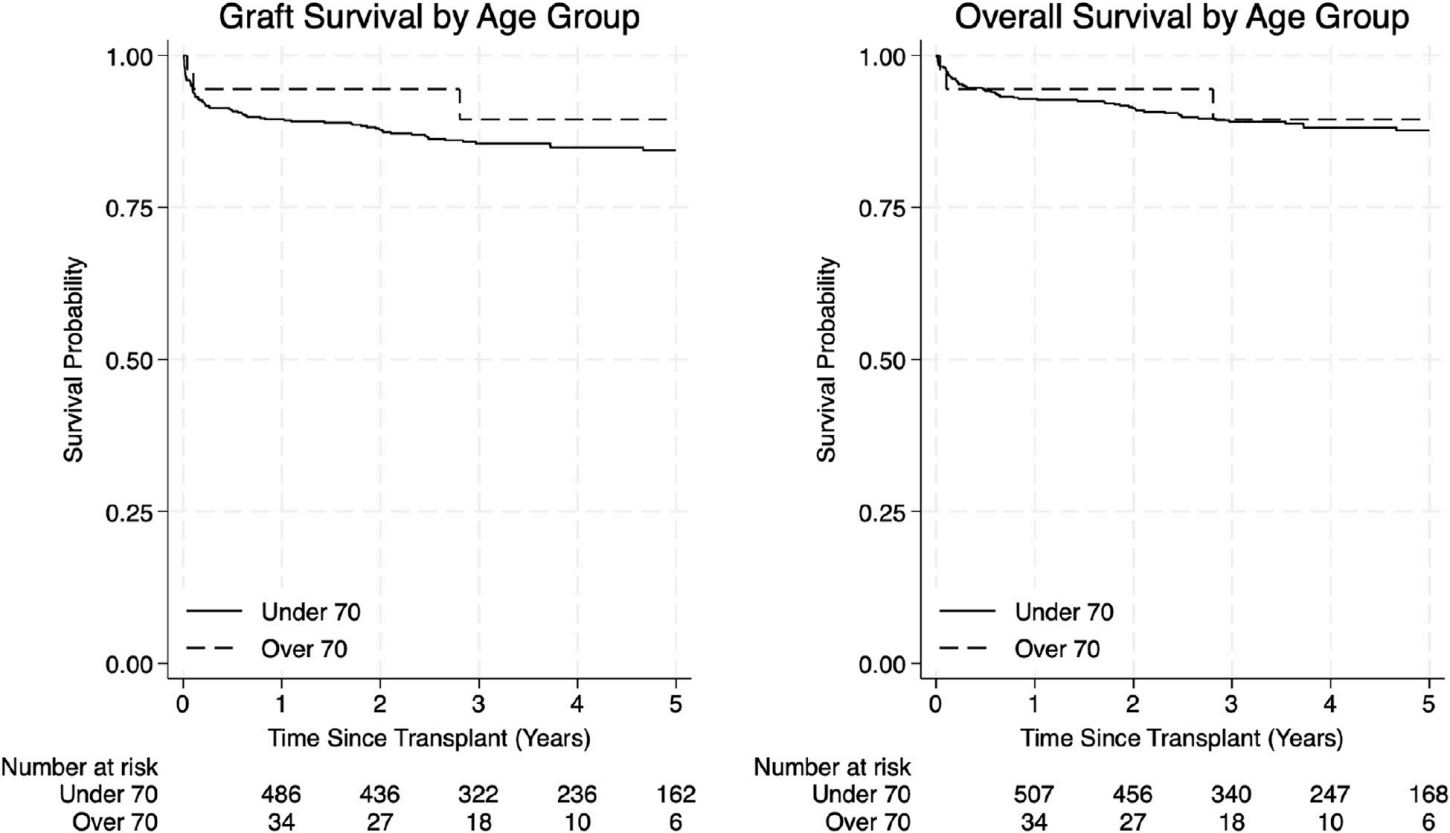
Graft survival and overall survival curves by age group.

In the elderly group, 1-year overall survival was 94.4% and 3- and 5-year survival was 89.5%. Graft survival was 94.4% at 1 year, and 89.5% at 3 and 5 years. In the younger group, 1-, 3- and 5-year overall survival were 92.9%, 89.1% and 87.7% respectively, while 1-, 3- and 5-year graft survival were 89.5%, 85.5% and 84.4%, respectively (Figure 2; Table 4).

**TABLE 4:** Overall survival and graft survival.

Graft survival				
Time (years)	Under 70	Over 70	Total	p-value
1	89.5 (86.6–91.8)	94.4 (79.6–98.6)	90.0 (87.2–92.2)	0.3484
3	85.5 (82.2–88.3)	89.5 (69.4–96.7)	86.0 (82.8–88.6)	
5	84.4 (80.9–87.4)	89.5 (69.4–96.7)	84.9 (81.5–87.8)	
**Overall survival**				
1	92.9 (90.4–94.7)	94.4 (79.6–98.6)	92.3 (90.6–94.8)	0.6947
3	89.1 (86.1–91.5)	89.5 (69.4–96.7)	89.2 (86.2–91.5)	
5	87.7 (84.3–90.4)	89.5 (69.4–96.7)	87.8 (84.6–90.4)	

Kaplan–Meier estimates of 1-, 3-, and 5-year graft survival, reported as Survival % (Lower CI – Upper CI). Comparison between groups was assessed using the Log-Rank test.

### Adjusted Outcomes With Case-Matched Analysis

A case-matched analysis was performed using matched cohorts created from propensity scores. The 3:1 matching algorithm identified two matched cohorts (n = 108 in the under 70 group and 36 in the over 70 group) in which all differences between recipient characteristics were balanced (Table 1). This allowed for the direct comparison of outcomes between the study cohorts, which again demonstrated similar perioperative outcomes, overall survival and graft survival. However, mCCI >1, which did not reach statistical significance in the unadjusted analysis, after matching was significantly higher in the elderly group compared to the younger cohort (19.4% vs. 10.9%, p = 0.045) (Table 1).

## Discussion

Our study shows that carefully selected LT recipients aged 70 years or older can achieve outcomes comparable to those of younger recipients, without significant differences in terms of postoperative complications, graft survival, and overall survival. The same results were obtained even after adjusted analysis eliminating preoperative differences, indicating that advanced age alone should not be considered an absolute contraindication to LT.

In recent years, LT in elderly recipients has become a prominent and pressing issue. In particular, the rising prevalence of MASLD and HCC in patients older than 65 years old has led to an increased demand for LT in this age group, and it has been demonstrated that this population can achieve promising results, reaching a 5-year overall survival of 77% [1]. However, the studies focusing on LT performed in recipients aged 70 or older are limited in the literature.

**FIGURE 3:**
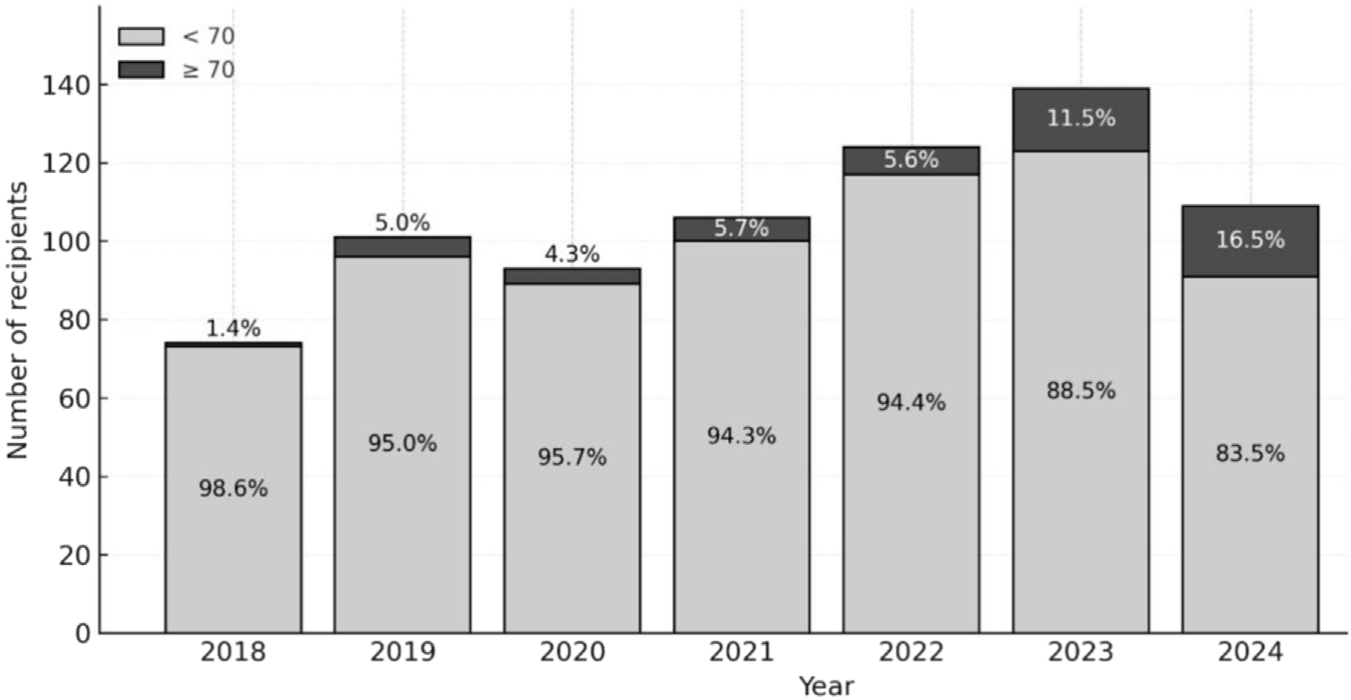
Proportion of Liver Transplant recipients Aged ≥70 by year from 2018 to 2024.

Accordingly, in our center the proportion of LT recipients aged ≥70 has increased from 1.4% in 2018 to 16.5% in 2024 (Figure 3).

Our recipients aged ≥70 years demonstrated favorable outcomes with an overall survival of 94.4% at 1 year and a 3-year and 5-year survival of 89.5% and graft survival of 94.4% at 1 year, and a 3-year and 5-year survival of 89.5%, with no difference compared to the younger cohort.

These results are consistent with those reported by Wilson et al. [12] in a multicenter cohort of 312 LT recipients aged ≥70 years, from 2007 to 2011, with a median MELD score of 15. They found a 1-year overall survival of 84.9% and similar postoperative complications, with no differences in the adjusted analysis compared to a matched cohort of patients younger than 70 years old.

However, their mid- and long-term results were less favorable, with unadjusted survival rates in the elderly cohort of 74.0% at 3 years and 64.1% at 5 years, significantly lower compared to younger recipients.

Similarly, a recent single-center study by Toiv et al. [13], reported outcomes, from 2014 to 2023, of 43 liver transplant recipients aged ≥70 years in comparison to a cohort of 956 recipients aged <70 years. The elderly group had a lower median MELD score (21.4 vs. 24.9), a higher prevalence of MASLD (51.2% vs. 23.0%) higher rates of diabetes and hyperlipidemia compared to the younger group. The elderly group had significantly longer median hospital stay (25.5 vs. 14 days), but there were no differences in overall patient or graft survival compared to the younger cohort. In the elderly group overall survival was 93% at 1 year, and 78% at 3 and 5 years. The outcomes of this study are similar to ours; however, our older cohort had lower median MELD score (13 vs. 21.4), and shorter hospital stay (14.5 vs. 25.5 days), with no difference between groups, likely as effect of selection.

Earlier series have reported similar outcomes (Table 5). A study from Mayo Clinic analyzing data from 1998 to 2004 showed comparable 5-year survival rates and no significant differences in postoperative complications between LT recipients aged ≥70 years (73%) and <60 years (76%), in two matched cohorts of 42 patients in each group [15].

**TABLE 5:** Overview of liver transplant outcomes in patients ≥70.

Author	Study population	No. of patients included	Compared groups	Outcomes
Lipshutz [14]	Single center, USA	926	≥70 years (n = 62) vs. 50–59 years (n = 864)	1-, 3-, 5- and 10-y OS: 73.3%, 65.8%, 47.1%, 39.7% (≥70 years); similar outcomes to younger patients in the unadjusted analysis
Aduen [15]	Single center, USA	84	≥70 (n = 42) vs. <60 years (n = 42)	Similar 5-year mortality risk and graft loss
Wilson [12]	SRTR and UHC databases, USA	12,445	≥70 years (n = 323) vs. 18–69 (n = 12,122) (1:1 matched)	Unmatched analysis: similar GS, worse 1-,3- and 5 years OS in the ≥70 years: 84.9, 74% and 64.1% Matched analysis: similar GS and OS
Sharpton [10]	UNOS registry	15,677	Stratified by age (18–59 (n = 11,966), 60–64 (n = 2,181), 65–69 (n = 1,177) and ≥70 years (n = 343) and MELD	≥70 years, MELD <20, 1 y GS: 85%, p = 0.01 ≥70 years, MELD 20–27, 1 y GS: 75%, p = 0.02 ≥70 years, MELD ≥28, 1 y GS: 56%, p < 0.001 GS significantly lower than other groups, however attenuated for LT-MELD <28
Sharma [11]	UNOS registry	61,873 (HCC groups) 47,043 (HCV groups)	≥70 years vs. <70 years (HCC vs. non-HCC and HCV vs. non-HCV)	5 years OS: 59.9% vs. 68.6% (HCC, p < 0.01) and 61.2% vs. 74.2% (non-HCC, p < 0.001) 5 years OS: 60.7% vs. 69% (HCV, p < 0.01) and 62.6% vs. 78.5% (non-HCV, p < 0.001)
Gil [26]	KNHI, Korea	9,415	≥70 years (n = 84) vs. younger groups	Risk of death in ≥70 years four-hold higher (OR = 4.1, 95% CI2.21–7.58; p < 0.001). 20% in-hospital mortality for ≥70 years
Toiv [13]	Single center, USA	999	≥70 n = (43) y vs. <70 years (n = 956)	Similar 1-, 3- and 5 years OS: 93, 78% and 78% (≥70 years) vs. 92, 86% and 82% (<70 years)

Similarly, Lipshutz et al., in a series from 1988 to 2005, demonstrated comparable graft and overall survival outcomes in patients aged ≥70 years (n = 62) compared to a younger group aged between 50 and 59 years (n = 864) [14]. Mousa et.al. reported a 5- and 10-year overall survival after LT of 70.8% and 43.6% for a cohort of 162 patients over 70 years old [24]. As previously reported [3], HCC did not affect the outcomes of elderly patients after LT.

The first concern in elderly recipients is represented by early mortality.

In a nationwide Korean study from Gil et al. [25], who analyzed 9415 LT from 2007 to 2016 and included 84 recipients older than 70 years (47 Deceased Donor Liver Transplant and 37 Living Donor Liver Transplant), reported that in-hospital mortality increased significantly with age beyond 60 years old. Specifically, post-liver transplant in-hospital mortality of recipients over 70, adjusted for baseline liver disease, was 4.2 times higher than in patients aged between 51 and 55 years (20.2% vs. 4.8%), and almost 3 times higher after adjusting for perioperative complications. They also reported increasing costs with age [25].

Similarly, Gomez Gavara et al. [6], indicated age over 65 years as an independent prognostic factor for mortality and graft loss. They analyzed data of 10,172 liver recipients (1,062 older than 65 years old) from European Liver Transplant Registry (ELTR) and showed worse overall patient survival in recipients over 65 years (85% at 1 year, 71% at 3 years, 59% at 5 years and 55% at 10 years) and worse graft survival (84% at 1 year, 71% at 3 years, 59% at 5 years and 55% at 10 years), confirmed after PSM. However, in their subgroup analysis, they identified transplant indication as a major prognostic factor in elderly patients. In this group, while outcomes for HCC were similar to younger patients, alcohol-related cirrhosis showed worse 5 and 10-year survival of 47% and 33%, attributed to frailty and sarcopenia.

In our study, due to accurate selection, no increased mortality was observed in the elderly group.

MELD score has a prominent influence on postoperative outcomes and remains an important variable in assessing candidacy for LT in patients over 70 years. Sharpton et al. [10] analyzed UNOS database that included all adult LT from 2005 to 2010, of which 343 were recipients older than 70 years old. They evaluated the combined effect of recipient’s age and MELD at the time of LT and reported a poor 1-year graft survival of 56% for recipients ≥70 and MELD ≥28.

However, in the same age group, MELD ≤20 and MELD between 20 and 27 showed better outcomes, with one-year graft survival rates of 85% and 75%, respectively, with no significant interaction between recipient age and MELD <28. Considering a lower median MELD in our older cohort, our results emphasize that, rather than age alone, liver disease severity should guide eligibility.

Moreover, cardiac risk is a critical prognostic factor in elderly recipients, as emerged by the series of Audet et al. [26]. In their study, recipients ≥65 years old (n = 34) showed higher mortality compared to younger controls. In the elderly group, 50% had a history of coronary artery disease (CAD), and the mortality rate due to cardiac causes was significantly higher. One and 3-year survival rates were 87% and 81.5%. As previously reported [14], cardiovascular events represent a major cause of mortality in older LT recipients and accurate cardiological screening is necessary to identify high-risk candidates.

In current literature, there are no strictly defined criteria for the exclusion of elderly patients from LT. Previous studies, as discussed above, have identified factors associated with increased post-transplant mortality risk: high MELD scores ≥28, the presence of CAD and high comorbidity burden, expressed as mCCI >1 [17].

At our center the selection process for elderly recipients is based on a case-by-case evaluation, taking into account the benefits and risks associated with transplantation, the general health status, comorbidities, and perioperative risk. However, as reflected by our elderly cohort characteristics, selection is oriented to minimize these high-risk factors. Accordingly, in our elderly cohort, only 2 patients had history of CAD and only 4 patients had MELD score ≥28. Moreover, our elderly cohort showed a low comorbidity burden, with 81.6% of the elderly patients having mCCI <2.

Several limitations must be acknowledged to interpret our results appropriately. First, this is a retrospective single-center analysis with a relatively small number of elderly recipients. Given the design of our study, our purpose was not to draw definitive conclusions but to contribute to the ongoing research with additional data from our experience. Additionally, as recipient selection was based on a multidisciplinary board evaluation, the process did not rely on objective or reproducible selection criteria. Although our outcomes are quite favorable, they may be the result of careful local selection practices and may not be reproduced by other centers.

Second, this study includes only patients who received LT and therefore does not account for patients aged ≥70 years who were evaluated but ultimately excluded from liver transplantation. Because data on declined candidates were not systematically recorded during the study period, we were unable to analyze the characteristics in this group and the reasons for exclusion.

Third, frailty and sarcopenia were not consistently assessed in our cohort and were therefore not included in the comparative analysis. The modified Charlson Comorbidity Index (mCCI) was used as a surrogate measure of comorbidity burden, but this does not replace validated frailty metrics. Sarcopenia, as a surrogate marker of frailty, has been associated with unfavorable outcomes following liver transplantation [22]; however, as shown in our results, variability in cut-off thresholds across studies limits its reliability as a standalone clinical parameter.

Finally, long-term outcomes such as 10-year overall and graft survival could not be evaluated, as the follow-up period was limited to 5 years.

These limitations highlight the need for prospective, multicenter studies with standardized data collection and reproducible selection criteria for elderly candidates; future research should incorporate sarcopenia in combination with objective and functional frailty assessments, evaluate both accepted and declined candidates, and include quality of life assessment [7] to better define transplant candidacy in patients ≥70 years.

## Data Availability

The raw data supporting the conclusions of this article will be made available by the authors, without undue reservation.
